# Characterizing growth and early hemodynamic inefficiencies: Phase-contrast magnetic resonance imaging analysis of the aorta in post-Fontan hypoplastic left heart syndrome

**DOI:** 10.1016/j.xjse.2025.100086

**Published:** 2025-12

**Authors:** Joao Filipe Fernandes, Hannah Bellsham-Revell, Prenali Dwisthi Sattwika, Mahta Ghahfarokhi, Hui Yann Yap, Anna Kiely, Xabier Morales Ferez, Oscar Camara, Julio Sotelo, James Wong, David Barron, Caner Salih, Kuberan Pushparajah, Adam J. Lewandowski, Pablo Lamata, Adelaide de Vecchi

**Affiliations:** a School of Biomedical Engineering and Imaging Sciences, Faculty of Life Sciences & Medicine, King's College London, London, United Kingdom; b Department of Congenital Heart Disease, Evelina London Children's Hospital, Guy's & St Thomas' Hospitals, London, United Kingdom; c Division of Cardiovascular Medicine, Radcliffe Department of Medicine, Oxford Cardiovascular Clinical Research Facility, University of Oxford, Oxford, United Kingdom; d Department of Internal Medicine, Faculty of Medicine, Public Health, and Nursing, Universitas Gadjah Mada, Yogyakarta, Indonesia; e Department of Engineering, Physense, BCN Medtech, Universitat Pompeu Fabra, Barcelona, Spain; f Departamento de Informática, Universidad Técnica Federico Santa María, Santiago, Chile; g Nuffield Department of Population Health, University of Oxford, Oxford, United Kingdom

**Keywords:** aorta, conduit function, hemodynamic inefficiency, 4D flow MRI, hypoplastic left heart syndrome, Fontan circulation

## Abstract

**Objective:**

To address the lack of routine hemodynamic assessment during aortic reconstruction in patients with hypoplastic left heart syndrome (HLHS) with a momentum-based metric from phase-contrast magnetic resonance imaging (MRI) data (4D Flow).

**Methods:**

4D Flow data were acquired in 100 subjects, including 22 Post-Fontan HLHS subjects (15 children, mean age 8.0 ± 1.7 years and 7 adolescents, mean age 15.1 ± 1.9 years) and 78 control subjects (mean age 7.6 ± 1.0 years). Comparative anatomic and functional analyses were performed in 4 segments: ascending aorta (AA), transverse arch (TA), and proximal and distal descending aortas (DA1 and DA2). Aortic growth was evaluated by its diameter indexed by body surface area, and early energy inefficiency was assessed by the advective pressure drop (PD_A_).

**Results:**

Post-Fontan HLHS children presented with early hemodynamic inefficiency at the TA–DA1 transition (median PD_A_ increment 81% larger in HLHS children than in controls; *P* = .01). The inefficiency in this region was further increased in the adolescent group (by an increment of 74% compared to the HLHS children; *P* = .022) despite the absence of anatomic obstruction and an average growth trajectory in line with that of controls.

**Conclusions:**

Early hemodynamic inefficiencies in the TA–DA1 transition in the HLHS aorta have been characterized. The PD_A_ metric allows for a noninvasive and robust characterization of inefficiencies with distinctive spatial and amplitude precision. Further studies will improve our understanding of the association between PD_A_ and outcomes.

## Graphical Abstract

**Figure:**
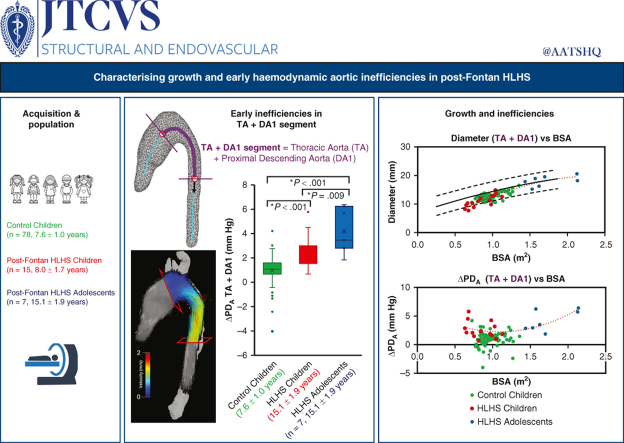

**Figure:**
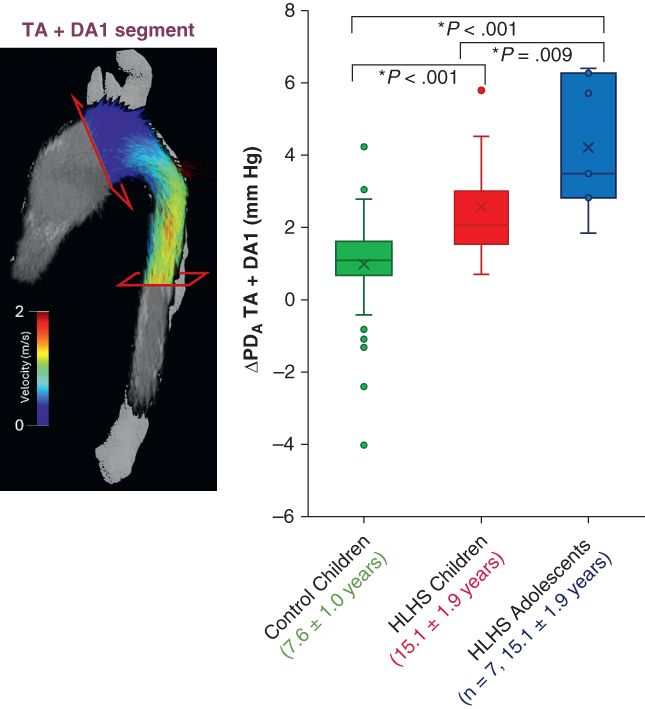
Key hemodynamic findings on post-Fontan coupling between reconstructed and native aorta.

**Central Message**

The advective pressure drop (PD_A_) derived from 4-dimensional flow MRI identified subtle early hemodynamic inefficiencies in coupling between Fontan-reconstructed aorta and native aorta in subjects with hypoplastic left heart syndrome.

**Perspective**

Standard routine tools do not fully unmask post-Fontan circulation inefficiencies. The use of advective pressure differences provides a detailed regional characterization of the reconstructed aorta and thus unique insights into functional single ventricles. The noninvasive approach allows continuous congenital heart disease monitoring. This study further informs age-related changes in post-Fontan hemodynamics.

Hypoplastic left heart syndrome (HLHS) is fatal without complex multistage surgery.^1^, ^2^, ^3^ The first stage of palliation enables unobstructed systemic blood flow by reconstructing the aortic arch using the main pulmonary artery. In the second stage, a superior cavopulmonary connection is created to offload the heart and ensure secure pulmonary blood flow, followed by completion of the total cavopulmonary connection to create the final Fontan circulation.^1^^,^^4^

Assessing the outcomes of each surgical stage and monitoring the growth of the aorta is critical, as any arch obstruction or inefficiency will have an impact on right ventricular and tricuspid valve function.^5^ With increasing numbers of survivors into adult life (65% total survival at a 5-year-follow-up),^6^ monitoring circulatory efficiency becomes important. Current methods for evaluating neo-aortic reconstructions include Doppler echocardiography, computed tomography, magnetic resonance imaging (MRI), and in selected cases, cardiac catheterization.^1^ MRI is mostly used to perform anatomic measurements, while hemodynamic assessment by either direct catheterization or Doppler-derived metrics provides key information on recoarctation, albeit with the limitations of invasiveness and potential suboptimal accuracy, respectively.^1^^,^^7^, ^8^, ^9^

Hemodynamic inefficiency, the viscous dissipation (VD) of energy by blood flow particles, is caused by the friction occurring when 2 particles travel adjacent to each other at different velocities or near a wall. VD is small in conditions of laminar (ie, “tidy”) flow and large in conditions of turbulent (ie, “chaotic”) flow. Various metrics based on blood flow velocity have been proposed to estimate this energy dissipation, for example, by computing the viscous losses directly or using a proxy metric, such as wall shear stress (WSS).^10^ These metrics require the computation of velocity gradients, the accuracy of which is highly dependent on the spatial resolution of the data and the fidelity of the segmentation.^11^ This makes energy dissipation extremely difficult to quantify in a robust and reproducible way, especially in the smaller infant vessels.

In this work, we circumvented this limitation by computing the maximum potential dissipation (ie, the momentum carried by the blood flow at each axial location) via quantification of the pressure differences driving the flow.^12^^,^^13^ Section 1 in the Appendix E1 provides a detailed explanation of this concept. This approach is followed in clinical guidelines for such conditions as aortic stenosis and obstructive hypertrophic cardiomyopathy. The specific metric in these cases is the “pressure gradient” (which should be called the “pressure difference” for physical accuracy) computed by the Simplified Bernoulli formula using Doppler-derived maximal velocity values.^14^

In the present work, we aimed to investigate how MRI-based analysis may offer novel insights into the hemodynamic efficiency in HLHS beyond the simplistic estimation from the Simplified Bernoulli equation, and to relate this to aortic growth. Specifically, we postulated that 4D Flow MRI can detect the presence of early signs of hemodynamic inefficiency, and we tested this hypothesis by comparing the profiles of efficiency along the whole aorta in HLHS patients with unobstructed aortic reconstruction and healthy age-matched subjects.

## Materials and Methods

### Patient Population and Surgical Procedure

**Figure 1:**
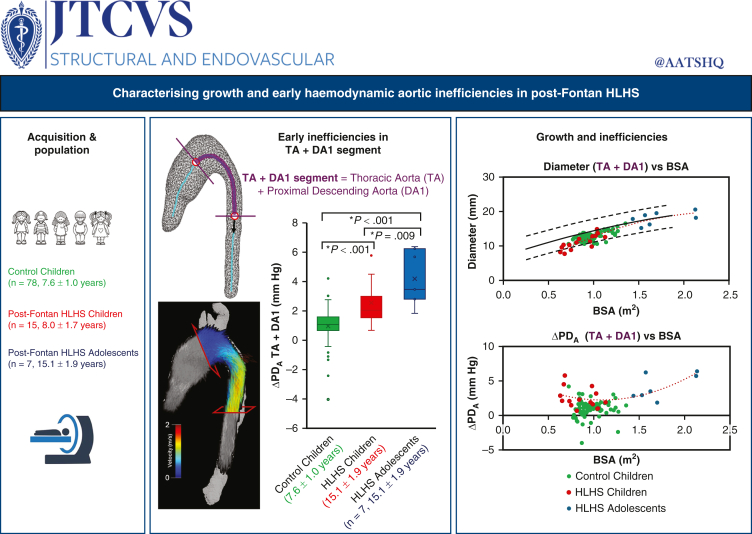
Graphical abstract. Inefficiencies, both geometric and hemodynamic, respectively characterized via diameter and advective pressure drop (ΔPD_A_) between transverse aortic arch (TA) and proximal descending aorta (DA1) increase in post-Fontan population relative to controls and increase with age. In the box-and-whisker plots with the box represented by median, 25th percentile, and 75th percentile, the whiskers represent the minimum and maximum values excluding outliers (individual points).

Retrospective datasets were divided into 3 groups (Table 1, Figure 1): healthy controls (n = 78; mean age, 7.6 ± 1.0 years), Fontan children (n = 15; mean age, 8.0 ± 1.7 years), and Fontan adolescents (n = 7; mean age, 15.1 ± 1.9 years). Healthy control datasets were acquired during the CHAPTER (Cardiovascular Health Assessment of Preterm and Term-born children) study (REC reference 18/WM/0131, the West Midlands Black Country Research Ethics Committee).^15^ A total of 155 children were recruited between 2019 and 2023, out of which 101 completed conventional cardiovascular MRI scans, including 78 healthy children with good-quality 4D Flow MRI data for aortic flow analysis. The exclusion criterion was evidence of congenital heart disease or significant chronic disease relevant to cardiovascular or metabolic status.

**Table 1:** Characteristics of the study groups

Characteristic	Control children	Post-Fontan HLHS children	Post-Fontan HLHS adolescents
Number	78	15	7
Age, y, mean ± SD	7.6 ± 1.0	8.0 ± 1.7	15.1 ± 1.9
Males/females, n	46/34	12/3	4/3
BMI, kg/m^2^, mean ± SD	16.4 ± 2.0	16.1 ± 1.6	22.7 ± 4.9
BSA, m^2^, mean ± SD	0.97 ± 0.13	0.86 ± 0.17	1.73 ± 0.29
Cardiac output, L/min, mean ± SD	3.7 ± 0.8	2.7 ± 0.7	6.8 ± 2.8
Heart rate, bpm, mean ± SD	84.2 ± 10.2	84.2 ± 6.4	72.8 ± 4.8
Use of general anesthesia	No	Yes	No

*HLHS*, Hypoplastic left heart syndrome; *BMI*, body mass index; *BSA*, body surface area.

The Fontan subjects were enrolled from routine monitoring at Evelina London Children's Hospital, London. Ethical approval was obtained from the NHS Health Research Authority (REC reference: 21/LO/0650, IRAS project ID: 304,329). Of the 33 original patients, 5 were scanned without 4D Flow acquisition, 4 were excluded because of incomplete or corrupted data, and 2 were excluded because of different anesthetic conditions compared to the rest of the group.

## Image Acquisition

### HLHS Cohort

MRI data were acquired on a Siemens Magnetom Sola 1.5-T or Philips Achieva 1.5-T machine. The 4D Flow data was acquired in free-breathing using a prospectively electrocardiography-triggered phase-contrast (PC) MRI sequence with the following parameters: velocity encoding of 150 cm/s, with 20 frames per cycle (corresponding to a temporal resolution of approximately 35 ms), spatial resolution of 2.0 mm isotropic voxels, and field of view of 160 × 80 × 40 mm. The parameters included a repeat time of 4.1 ms, echo time of 2.8 ms, flip angle of 7°, and bandwidth of 500 Hz. Respiratory gating was implemented for motion correction, and data were reconstructed using an in-house k-t principal components analysis method.^16^ Automatic eddy current correction was applied to all datasets. The HLHS children were scanned under general anesthesia, while the HLHS adolescents were scanned while awake.

### Healthy Age-Matched Cohort

CHAPTER participants underwent MRI on a 3-T PRISMA scanner (Siemens) without anesthesia. The 4D Flow data were acquired using a free-breathing, retrospective ECG-triggered and respiratory-gated sequence,^17^^,^^18^ with a velocity encoding of 120 cm/s, temporal resolution of 38.88 ms, and spatial resolution of 2.2 mm (isotropic voxels). Other parameters included a repeat time of 4.9 ms, echo time of 2.65 ms, and a flip angle of 7°.

## Imaging Data Analysis

### Image Preprocessing

Segmentation was performed on the reconstructed PC-MRI images at peak systole using a semiautomated approach based on U-Net.^19^ A time-averaged PC-MRI angiogram was generated by combining both the magnitude and phase images.^20^ A small dataset comprising 10 cases was segmented manually^21^ and served as the training set for the neural network. Subsequently, segmentations of the remaining cases were inferred by the U-Net and visually inspected for errors. Additional manual corrections were performed in the more challenging HLHS anatomies.

The aorta was then divided into 4 segments: ascending aorta (AA), transverse arch (TA), and proximal and distal thoracic descending aortas (DA1 and DA2). The segments were delineated in a semiautomated fashion using the following anatomic landmarks: aortic valve plane, brachiocephalic artery, left subclavian artery, level of the descending aorta aligning with the aortic valve, and diaphragm level.^21^ The combined transition from the reconstructed TA segment to the DA1 segment was also considered (Figure 1).

### Geometric Analysis: Curvature and Diameter

Aortic diameter was computed at each point along the center line as the average diameter of the circle intersecting the 3D surface model and a plane perpendicular to the center line.^22^ Values were then normalized by body surface area (BSA). Aortic curvature was assessed by the inverse radius of the circle defined by 3 sequential points along the center line.

### Functional Analysis

Blood velocity vectors were reconstructed from 4D Flow data using GT-Flow v4.9.0 (GyroTools) and used to characterize conduit function (ie, the ability of the system to transport flow efficiently) by computing the advective pressure drop (PD_A_). This metric quantifies the pressure difference needed to create the observed flow momentum at each location along the vessel length.^12^ As such, the PD_A_ captures the effect of the interplay between vessel geometry (ie, tapering, sharp diameter, or curvature changes) and circulatory demand (ie, flow rate).^13^

The conduit function was computed at peak systole when the momentum was maximum.^22^ The PD_A_ was assessed by the simplified advective work–energy relative pressure (SAW) formulation.^12^ A correction of the widely used Simplified Bernoulli, SAW quantifies the spatial acceleration needed to accommodate a given flow rate through a given vessel caliber assuming an initial null momentum.^12^^,^^14^ By accounting for the complete flow distribution in a vessel cross-section instead of simply using the peak velocity like in Simplified Bernoulli, the SAW approach is more precise and accurate.^12^

Functional inefficiencies are identified as either a peak value of PD_A_ at a given location (eg, due to stenosis) or a PD_A_ increment (ΔPD_A_) across a given vascular segment (eg, due to gradual vessel tapering or a mismatch between flow rate and vessel size). Both metrics were benchmarked against the values observed in healthy age-matched subjects (baseline).

### Statistical Analysis

Continuous variables are expressed as mean ± standard deviation (SD). The Shapiro-Wilks test was used to assess the normality of all metrics. Mean values were compared using the 2-sided Student *t* test for normally distributed variables and the Mann-Whitney *U* test for variables that did not follow a normal distribution, with a 95% significance level (*P* < .05). Box-and-whisker plots for the study groups were generated with the lower, mid and upper lines of the box representing the lower quartile (25th percentile), the median, and the upper quartile (75th percentile). The lower and upper whiskers represent the minimum and maximum values of nonoutliers, extra dots represent outliers, and the “X” indicates the mean value. All analyses were performed with SPSS v29.0.2.0 (IBM).

## Results

### Comparison of HLHS Children and Age-Matched Controls

**Figure 2:**
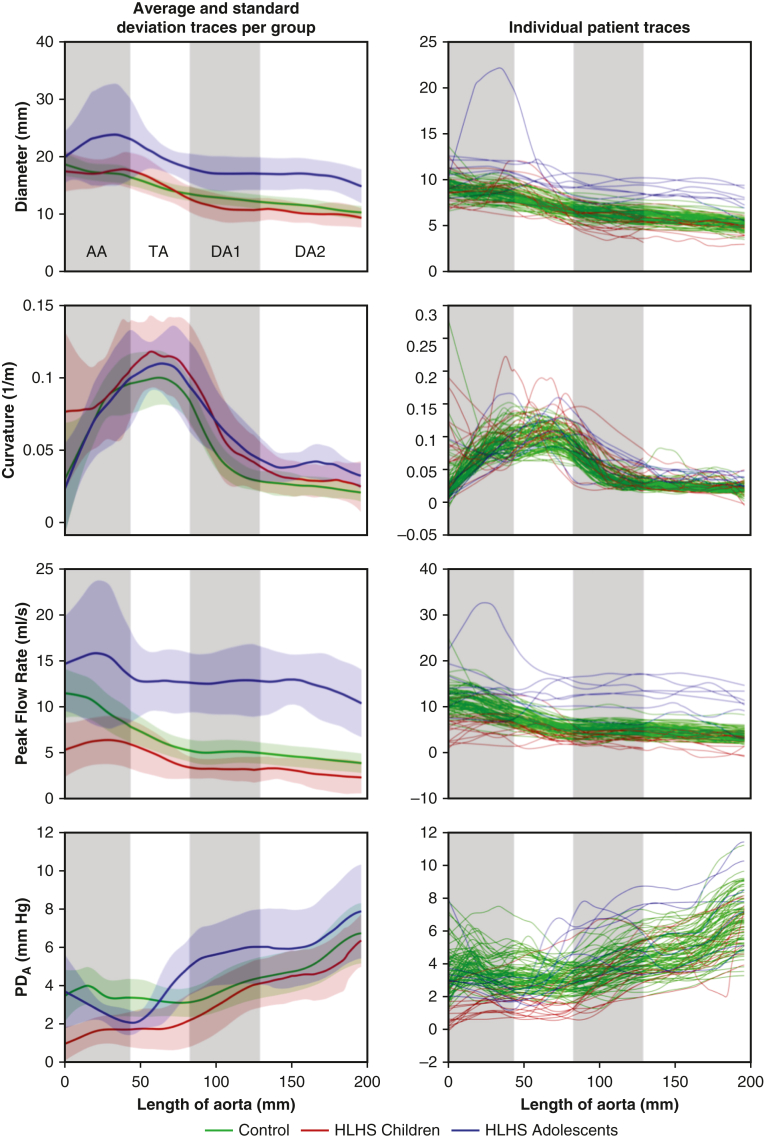
Comparative geometric and hemodynamic traces along aortic center line for the 3 study groups, including mean ± standard deviation (*left side*) and individual traces (*right side*). The overall aortic length is the average length of the population, and each individual trace is fitted to the average length of each of the 4 regions: ascending aorta (AA), transverse aortic arch (TA), and proximal and distal descending aortas (DA1 and DA2). Note that this figure shows total advective pressure drop (PD_A_) variations along the center line, and the increment per region (ΔPD_A_) would be the change of amplitude in each region considered.

The reconstructed aortas in the HLHS children had nonsignificantly different diameters compared to controls and to HLHS adolescents (Table 2). A larger interindividual variability in curvature was observed in the HLHS cohort, with a higher proportion of “gothic” arches^23^ with curvature values rising sharply at the TA (Figure 2). No other significant differences in geometry were observed in the other aortic segments.

**Table 2:** Regional aortic anatomic and hemodynamic results

Segment	Measurement	Control (N = 78), mean ± SD	HLHS children (N = 15), mean ± SD	HLHS adolescents (N = 7), mean ± SD	*P* value, control vs HLHS children	*P* value, HLHS (children vs adolescents)
AA	Diameter/height^2.7^, mean (mm/m^2.7^)	9.23 ± 1.27	10.73 ± 4.45	8.42 ± 3.91	.218	.239
	Curvature, peak (L/mm)	0.10 ± 0.03	0.09 ± 0.03	0.17 ± 0.08∗	.577	.041
	Flow rate/BSA, mean, (L/min/m^2^)	10.35 ± 1.5∗	7.17 ± 2.80	8.78 ± 3.067	<.001	.264
	PD_A_, peak (mm Hg)	3.64 ± 0.89∗	1.97 ± 1.04	3.73 ± 1.94	<.001	.056
	ΔPD_A_, maximal increment (mm Hg)	−0.08 ± 1.41∗	0.75 ± 0.86	−1.63 ± 2.32∗	.005	.034
TA	Diameter/height^2.7^, mean (mm/m^2.7^)	7.86 ± 1.11	9.38 ± 3.96	7.41 ± 2.90	.161	.206
	Curvature, peak (L/mm)	0.10 ± 0.02∗	0.11 ± 0.02	0.22 ± 0.09∗	.099	.015
	Flow rate/BSA, mean (L/min/m^2^)	6.57 ± 1.03	5.41 ± 2.11	7.40 ± 1.37∗	.054	.017
	PD_A_, peak (mm Hg)	3.61 ± 0.95∗	2.41 ± 1.23	5.02 ± 1.56∗	.002	.003
	ΔPD_A_, maximal increment (mm Hg)	−0.24 ± 0.81∗	0.48 ± 1.20	2.91 ± 1.65∗	.041	.007
DA1	Diameter/height^2.7^, mean (mm/m^2.7^)	6.75 ± 0.97	6.65 ± 2.08	6.65 ± 2.08	.861	.310
	Curvature, peak (L/mm)	0.08 ± 0.02	0.09 ± 0.03	0.16 ± 0.07∗	.814	.025
	Flow rate/BSA, mean (L/min/m^2^)	6.64 ± 0.59	6.45 ± 0.75	5.28 ± 0.71∗	.358	.004
	PD_A_, peak (mm Hg)	3.67 ± 0.92	4.10 ± 1.26	6.16 ± 1.70∗	.222	.018
	ΔPD_A_, maximal increment (mm Hg)	1.28 ± 0.87	1.96 ± 1.36	1.05 ± 1.77	.083	.259
DA2	Diameter/height^2.7^, mean (mm/m^2.7^)	5.68 ± 0.82	4.26 ± 2.71	3.98 ± 2.12	.063	.799
	Curvature, peak (L/mm)	0.03 ± 0.01	0.04 ± 0.02	0.09 ± 0.03∗	.137	.002
	Flow rate/BSA, mean (L/min/m^2^)	5.86 ± 0.54	5.79 ± 0.86	5.08 ± 0.68	.527	.105
	PD_A_, peak (mm Hg)	6.73 ± 1.57	6.36 ± 1.38	7.87 ± 2.45	.400	.170
	ΔPD_A_, maximal increment (mm Hg)	2.31 ± 1.03	2.26 ± 1.11	1.83 ± 1.58	.874	.541

The aorta was sectioned in 4 regions: ascending aorta (AA), transverse aortic arch (TA), and proximal and distal descending aortas (DA1 and DA2). The aortic valve plane distinguishes DA1 and DA2. Three participant groups were considered: healthy control children, post-Fontan hypoplastic left heart syndrome (HLHS) children age 6 to 10 years, and post-Fontan HLHS adolescents age 12 to 18 years.

*BSA*, Body surface area; *PD*_*A*_, advective pressure drop.

∗ Independent-samples *t* test (2 tests): indicates significant differences compared to the HLHS children cohort (*P* < .05).

**Figure 3:**
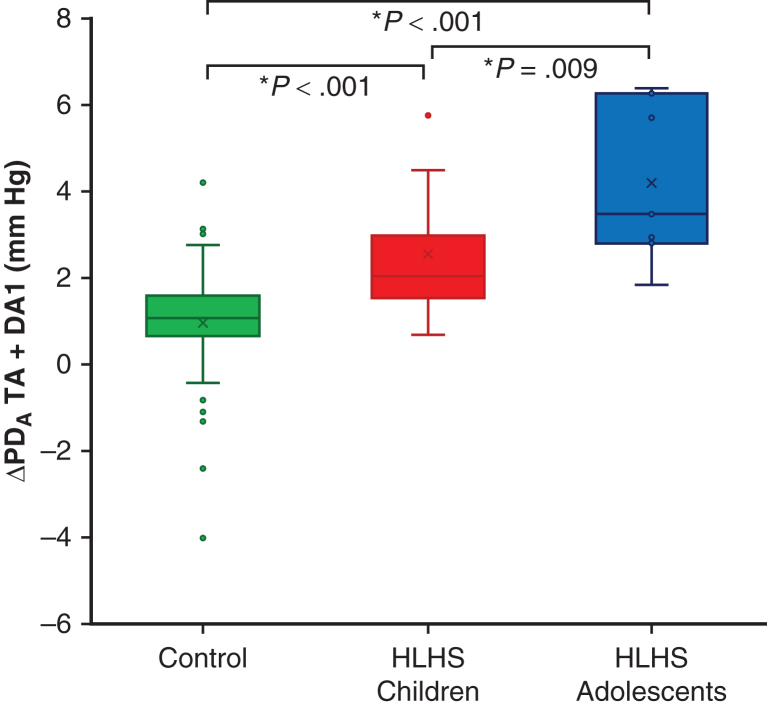
Box-and-whisker plots for advective pressure drop increment (ΔPD_A_) over the segment composed by transverse arch and proximal descending aorta (TA + DA1) in each study group. In the box-and-whisker plots with the box represented by median, 25th percentile, and 75th percentile, the whiskers represent the minimum and maximum values excluding outliers (individual points). ∗Significantly different *P* value > .05. The “X” represents the mean value per subgroup.

In terms of conduit function, the HLHS children displayed lower mean peak PD_A_ in the AA and TA reconstructed segments compared to controls, despite having comparable diameters (AA: 2.0 ± 1.0 mm Hg vs 3.6 ± 0.9 mm Hg; TA: 2.4 ± 1.2 mm Hg vs 3.6 ± 1.0 mm Hg; *P* < .002). No statistically significant differences in PD_A_ values were observed over the rest of the aorta; however, HLHS children had significantly higher ΔPD_A_ values across the combined TA-DA1 segment compared to healthy controls (2.3 ± 1.4 mm Hg vs 1.0 ± 1.2 mm Hg; *P* = .003), as shown in Figure 3.

Finally, the flow rate, normalized to BSA, was significantly smaller in HLHS children compared to controls in the AA (*P* < .001), but not in the remaining segments. Furthermore, in HLHS adolescents, the mean normalized flow rate was significantly higher in the TA but significantly lower in the DA1 compared to the HLHS children (TA: 5.4 L/min/m^2^ ± 2.1 vs 7.4 ± 2.9 L/min/m^2^, *P* = .017; and DA1: 6.5 ± 0.8 L/min/m^2^ vs 5.3 ± 0.7 L/min/m^2^, *P* = .004).

### Adolescent HLHS Patients and Inspection of Individual Cases

**Figure 4:**
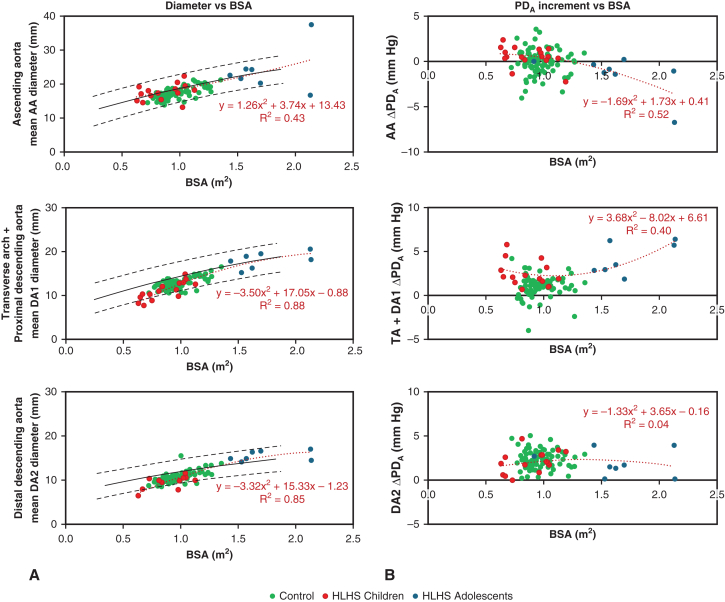
Post-Fontan hypoplastic left heart syndrome (HLHS) changes in geometry and hemodynamics versus controls. A, Mean diameter versus body surface area (BSA) per study group in each aortic region. Reference values in children and adolescents as described by Kaiser and colleagues^24^ (average and 95% confidence intervals) are shown in *black*.^24^ B, Increment of advective pressure drop (PD_A_) per aortic region (ΔPD_A_) versus BSA.

Characteristics of the 2 Fontan groups are compared in Table 2. Flow rate was significantly higher in HLHS adolescents compared to HLHS children in all segments (overall, *P* ≤ .011), while the peak PD_A_ was significantly higher in the TA and DA1 only and the ΔPD_A_ was significantly higher in the AA and the TA only. Furthermore, the aortic growth in HLHS children and adolescents was within the healthy normal range in the literature (Figure 4, *A*)^24^; however, in the HLHS adolescents, the ΔPD_A_ showed a sharp increment with BSA in the TA-DA1 region. In addition, a decreasing trend in ΔPD_A_ was observed along the AA (created by an outlier with an aneurysm in the AA), while negligible variation was observed in the DA2 (Figure 4, *B*).

**Figure 5:**
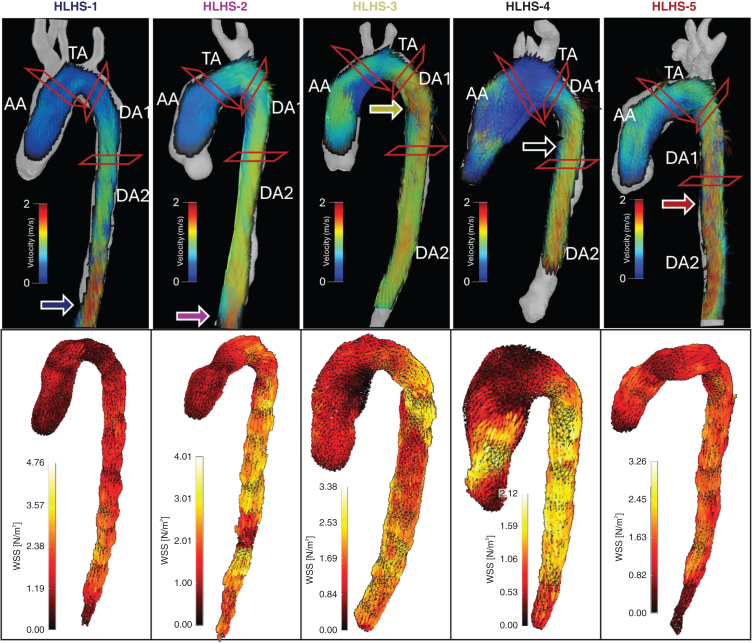

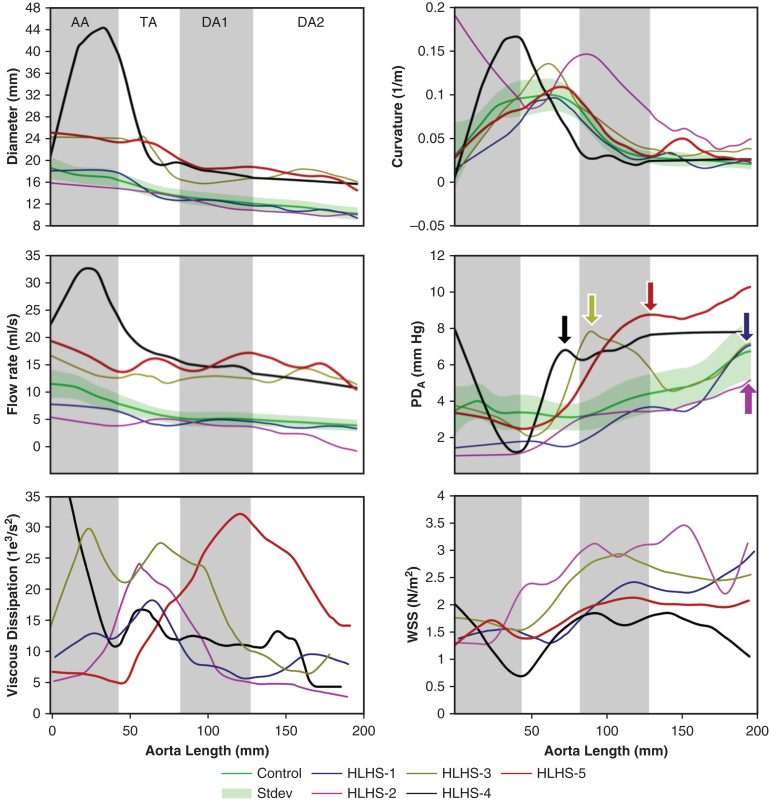
Five examples of post-Fontan patients with different anatomic and functional profiles. (*Top*) Velocity vector fields (*upper*) and wall shear stress (WSS) magnitude (*lower*) overlaid onto the aortic segments. (*Bottom*) Plots showing variations in anatomic and functional biomarkers along the aorta. The *colored arrows* indicate the location of peak PD_A_ for selected patients.

Anatomic and functional metrics exhibited large interindividual variability in the HLHS children (see the individual traces in Figure 2). This variability was further amplified in the HLHS adolescents. To illustrate the mechanisms driving this variability, we selected 5 cases with salient characteristics in terms of peak curvature, sudden transitions of diameter, or sudden increments of PD_A_ (ie, nascent hemodynamic inefficiency). These 5 cases are illustrated in Figure 5, which also compares the inefficiency metrics WSS and VD.

• Case HLHS-1 (blue line, age 10 years) had geometric and functional metrics roughly matching the characteristic of the control group along the entire aortic length. The main differentiating characteristic of this case was a more severe reduction of diameter along the TA compared to the mean cohort value, a feature commonly observed in reconstructed HLHS aortas. However, this reduction did not affect conduit function, with no significant increments in PD_A_ observed in this region.
• Case HLHS-2 (pink line, age 6 years) presented an abnormal curvature profile, with a “gothic” arch at the TA–DA1 transition. Despite this sharp curvature, the aorta was able to accommodate the momentum generated by the ventricle without conduit inefficiencies (no corresponding increment in PD_A_ over this region). This case had the highest WSS values (but not the largest VD), possibly due to the combination of small size and curvature effects, leading to a skewed velocity profile.
• Case HLHS-3 (yellow line, age 17 years) was selected for its abrupt reduction in diameter at the TA, which results in a colocalized increase in WSS and an abrupt increment in PD_A_ (5.7 mm Hg), signaling the presence of functional inefficiency.
• Case HLHS-4 (black line, age 16 years) had a huge diameter reduction at the TA from an aneurysm at the AA. This diameter reduction was associated with an abrupt increment of PD_A_ (6.7 mm Hg), signifying impaired conduit function. In this case, this inefficiency was sustained and slightly increased throughout the whole DA, where the aorta showed tapering. This effect was not reflected in WSS and VD values.
• Case HLHS-5 (in red, age 15 years) was another example of a large increment in PD_A_ over the TA–DA1 transition (6.3 mm Hg). In this case, however, this ΔPD_A_ corresponded to a mild decrease in diameter and a TA curvature in the range of control subjects. The PD_A_ was maximal at the end of the TA, where the VD also peaked, while the WSS was comparable to that of HLHS-1. Note also that the PD_A_ change was less abrupt than in the previous 2 cases and spanned a greater vascular length.

## Discussion

A noninvasive hemodynamic assessment of the aorta has been proposed to identify functional inefficiencies along the aortic length in Fontan patients. Energy inefficiencies were observed in the critical region where the reconstructed TA joins the native DA1. This was enabled by the precision of the PD_A_ metric based on 4D Flow MRI, which can detect minimal differences in conduit function across patient groups even with small sample sizes.

In addition, the PD_A_ metric allows characterization of the spatial distribution of the momentum that will lead to dissipation, and with it the interplay between aortic anatomic features and flow demand that underpins the hemodynamic inefficiencies. Furthermore, this cross-sectional study offers insights into age-related hemodynamic changes in the reconstructed arch in Fontan patients.

### What Makes a “Good” Aortic Reconstruction in HLHS?

A good aortic reconstruction is one that achieves a satisfactory conduit function through sufficiently wide vessels and smooth transitions in diameters. The PD_A_ metric can detect when vessels are not wide enough (ie, large peak PD_A_) or when transitions are abrupt (ie, large ΔPD_A_). The averaged spatial profiles of conduit function in Figure 2 indicate that the reconstructed aortas in HLHS children show no anatomic obstructions or severe hemodynamic inefficiencies compared to age-matched controls.

The AA and TA diameters were nonsignificantly different between HLHS patients and age-matched controls. Nevertheless, peak PD_A_ values in these segments were lower in the HLHS patients compared to controls (Table 1), consistent with our previous 4D Flow analysis in pre-Fontan HLHS children with a mean age of 2.8 ± 0.7 years.^22^ Therefore, in the age ranges of childhood and adolescence studied here, the surgical reconstructions analyzed can be characterized as satisfactory, suggesting no need for larger diameters as reported in other studies.^5^

However, relatively larger increases in PD_A_ along the TA–DA1 transition were observed in HLHS aortas compared to controls, suggesting the presence of nascent hemodynamic inefficiencies even under anesthesia that possibly could be exacerbated under stress.^25^ The challenge in aortic reconstructions lies in this TA–DA1 transition, where a smooth change in diameter between the enlarged segment and the native DA is desirable. Our results show that this smooth transition is not always maintained through the development stages, as demonstrated by the presence of early flow inefficiency along the TA-DA1 segments of Fontan children compared to healthy controls. This finding agrees with our previous study in pre-Fontan patients, which identified the transition to the DA segment as the weak functional link.^22^

A larger diameter in the reconstructed AA will make the TA–DA1 transition more challenging, because it will need to accommodate a larger change of vessel caliber. However, the onset of early inefficiencies in the TA–DA1 transition might not have resulted solely from surgical factors; it also may have developmental causes and/or share mechanistic explanations with the etiology of coarctation of the aorta, for which hemodynamic and cellular migration factors have been proposed and studied.^26^ Moreover, these early inefficiencies may have a downstream impact on aortic aneurysm formation in this region, as shown previously.^27^

### Interplay Between Aortic Anatomy and Inefficiencies

A localized increase in PD_A_ enables the patient-specific detection of nascent aortic inefficiencies, and our data suggest that curvature and diameter changes are the 2 main factors. The mechanism of the sharp reduction of diameter is the need for smaller vessel calibers to accommodate the flow rate at the expense of a pressure drop. In a gothic arch, characterized by a localized large curvature value, the mechanism is the onset of flow separation and associated losses.^28^

When present in our cases, hemodynamic impairment is mainly colocated with a diameter reduction rather than with sharp curvatures in the aortic anatomy, such as those seen in gothic arches (eg, HLHS-3 and HLHS-4 in Figure 5). Nevertheless, a single anatomic metric, such as diameter or curvature, is not always able to identify areas of increased inefficiency. Case HLHS-5 is a good illustrative example, in which neither the peak curvature nor the diameter reduction is a plausible explanation for the conduit inefficiency.

Inefficiency not only is dictated by the anatomic features, but also is mediated by the circulatory demand, that is, the flow rate. The same anatomy might be able to accommodate efficiently a range of flow rates but drastically lose conduit ability when this range is exceeded, as illustrated by the variability of metrics in HLHS patients. Flow demand can lead to acute increases in cardiac output, for example, under physiologic stress.^5^ Therefore, it is crucial to consider aortic inefficiencies as a dynamic process that depends on the interplay between flow demand and anatomic features in the aorta, both of which are linked to growth.

The study of localization and magnitude of hemodynamic inefficiencies also has been addressed by the complementary analysis of downstream VD. The challenge lies in assessing turbulence, which is responsible for most of the dissipation, and both simulation studies and experimental approaches with specialized MRI sequences have been proposed.^29^^,^^30^

Further research is needed to ascertain the relationships between these local aortic metrics and other physiologic aspects, such as the energetics and efficiency of ventricular contraction, the systemic BP response, near-wall turbulence, and the patient's exercise tolerance. Understanding the interplay of mechanisms will inform surgical strategies and risk stratification of patients.

### What Makes a “Good” Efficiency Metric?

Hemodynamic energy losses occur at very fine scales, and the PD_A_ metric is a surrogate for inferring these losses. We claim that PD_A_ is an adequate choice to assess inefficiency because of its robustness compared to alternative metrics (Appendix E1, Section 2) and its unprecedented ability to detect subtle hemodynamic inefficiencies early.

The PD_A_ provides complementary information for alternative metrics of flow inefficiency, such as WSS and VD. As illustrated in Figure 5, a high PD_A_ in the descending aorta does not colocate with high WSS or VD. The PD_A_ computes the maximum potential dissipation (Appendix E1, Section 1) without computing spatial derivatives as in WSS or VD computations. As such, it is a much more robust approach, especially in pediatric cases with congenital heart disease, where image resolution is challenging and accurate diagnosis crucial.

### Not All Pressure Metrics Are the Same

The TA–DA1 transition is an important anatomic aspect in the reconstruction of hypoplastic aortas. The magnitude of the pressure drops in the TA–DA1 transition is on average 2.43 mm Hg in the HLHS children, reaching peaks of 6 to 7 mm Hg in some individual cases (Figure 5). These values are smaller compared with, for example, the average of 36 mm Hg reported in adult aortic coarctation^31^ or the threshold of 20 mm Hg defined in guidelines for treating aortic coarctation.^32^ In these comparisons, there are important methodologic considerations, as discussed next.

First, echocardiography-based Simplified Bernoulli formulation overestimates the pressure differences up to a factor of 2, as flow is simplified to a single streamline with a single peak velocity.^14^ The full velocity profile is needed to get an accurate metric of PD_A_, and this can be achieved by the MRI-based SAW formulation of this study. Section 2 of the Appendix E1 described illustrative quantitative differences in silico and in patients.^12^ As such, a threshold of 20 mm Hg in guidelines for coarctation of the aorta based on Simplified Bernoulli approximately corresponds to a threshold for SAW-based PD_A_ of 13 mm Hg using the corrective factor of 0.65 estimated for aortic stenosis.^12^

The second consideration is that catheter recordings capture the peak-to-peak pressure difference, not the actual instantaneous pressure difference captured by velocity-based metrics, such as Simplified Bernoulli and SAW approaches.^14^ The correction of the Simplified Bernoulli equation by the proximal velocity (ie, the modified Bernoulli equation) has been shown to improve the matching of catheterized and echocardiography-based assessment of obstructions.^31^ This correction in our study is conceptually the use of ΔPD_A_ along the TA-DA1 segment.

### Route for Clinical Impact

This work proposes a noninvasive method for characterizing flow inefficiencies with an unprecedented ability to assess their location and magnitude. Clinical researchers can now use this method and advance our understanding of the onset and progression of flow obstructions. The significance is that we can advance in this direction, and eventually in the management of congenital vascular conditions that experience obstructions, without the barriers of current gold standard of catheterized pressure sensors. The ethical barriers due to the interventional risks are lowered, as are the costs. More frequent and more insightful (ie, with accurate location and magnitude) examinations of flow efficiency are possible with an imaging-based technique.

This work is a proof-of-concept study that cannot be immediately translated into any clinical management decision. We envision 2 avenues of improvement in clinical practice. First, vascular reconstructive surgeries will improve from the ability to characterize their outcomes with unprecedented detail. In this direction, our results on the reconstruction of the hypoplastic aorta provide the benchmark of key metrics to characterize the outcomes of the surgery in its most challenging aspect, the TA–DA transition. Similar characterizations of reconstructed vascular segments and connections, such as those involved in the Fontan circulation, are now possible. The second avenue will be the definition of optimal and personalized follow-up practices in the management of congenital conditions, along with the early detection of risk of obstruction, such as the onset of coarctation of the aorta. Longitudinal studies are first needed to characterize the onset and progression of obstructions.

### Limitations

The experimental protocols (4D Flow MRI acquisitions and use of anesthesia) differed between the HLHS and control groups. While differences in 4D Flow acquisitions do not affect the computation of PD_A_, the difference in anesthesia use is a major limitation of this work. Its use in the HLHS children is interpreted to be the main cause of the lower peak flow and cardiac output (Table 1).

Controlling for the impact of anesthesia is challenging. Among other physiologic factors, the interplay between flow demand and advection (ie, PD_A_) is not linear (ie, half flow demand will not translate into half PD_A_). In any case, a decrease in flow demand that may be caused by anesthesia will inherently lead to a reduction in momentum. Therefore, the loss of conduit function in HLHS signaled by the higher ΔPD_A_ might in fact become amplified once the child returns to normal awake conditions. This then might explain the larger ΔPD_A_ observed in the HLHS adolescents compared to the HLHS children shown in Figure 3.

Our initial attempt to study the potential impact of anesthesia with our population sample is reported in the Appendix E1, Section 3. Ultimately, the claims of the metrics of HLHS subjects matching those of controls are limited due to this experimental bias. Inefficiency at the TA–DA1 transition may be greater in the awake condition, and the significance of this metric of hemodynamic inefficiency may be greater. Furthermore, there is also bias in the control group, with no matched adolescents.

## Conclusions

PC-MRI allows a quantitative and comprehensive functional characterization of the neo-aorta. A metric of conduit function, the PD_A_, is proposed to provide a precise location and magnitude of inefficiencies arising from the interplay between flow demands and anatomy. As a completely noninvasive metric, the PD_A_ allows more frequent and targeted hemodynamic assessments of aortic function in a young and often fragile cohort of patients, overcoming the limitation of discrete spatial measurements at restricted locations intrinsic to catheter measurements.

## Conflict of Interest Statement

PL reported a Wellcome Trust Senior Research Fellowship (209450/Z/17/Z). All other authors reported no conflicts of interest.

The *Journal* policy requires editors and reviewers to disclose conflicts of interest and to decline handling or reviewing manuscripts for which they may have a conflict of interest. The editors and reviewers of this article have no conflicts of interest.

## Figures and Tables

**Figure E1:**
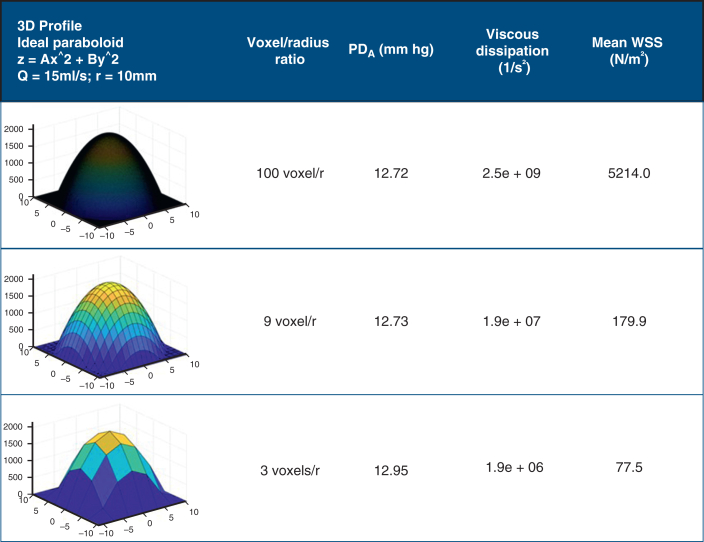
In silico comparison of dependency on spatial resolution of 4D Flow inefficiency metrics, including momentum quantification via advective pressure drop (PD_A_) quantification,^12^ wall shear stress (WSS), and viscous dissipation (VD).

**Figure E2:**
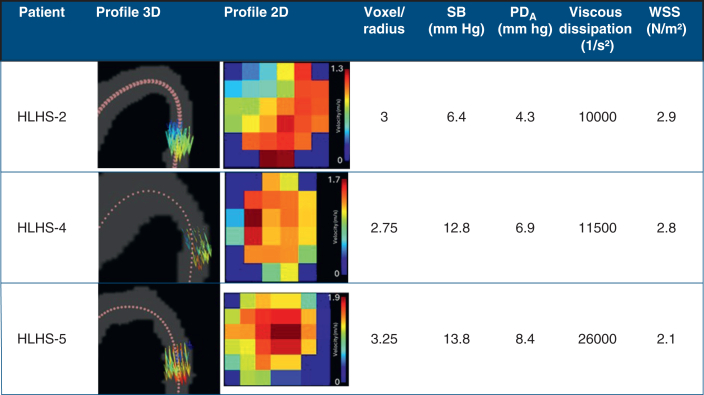
Comparison of blood flow profile and inefficiency metrics at the transition point between the transverse arch and the descending aorta in 3 selected cases. *SB*, Simplified Bernoulli pressure drop; *PD*_*A*_, advective pressure drop; *WSS*, wall shear stress.

**Figure E3:**
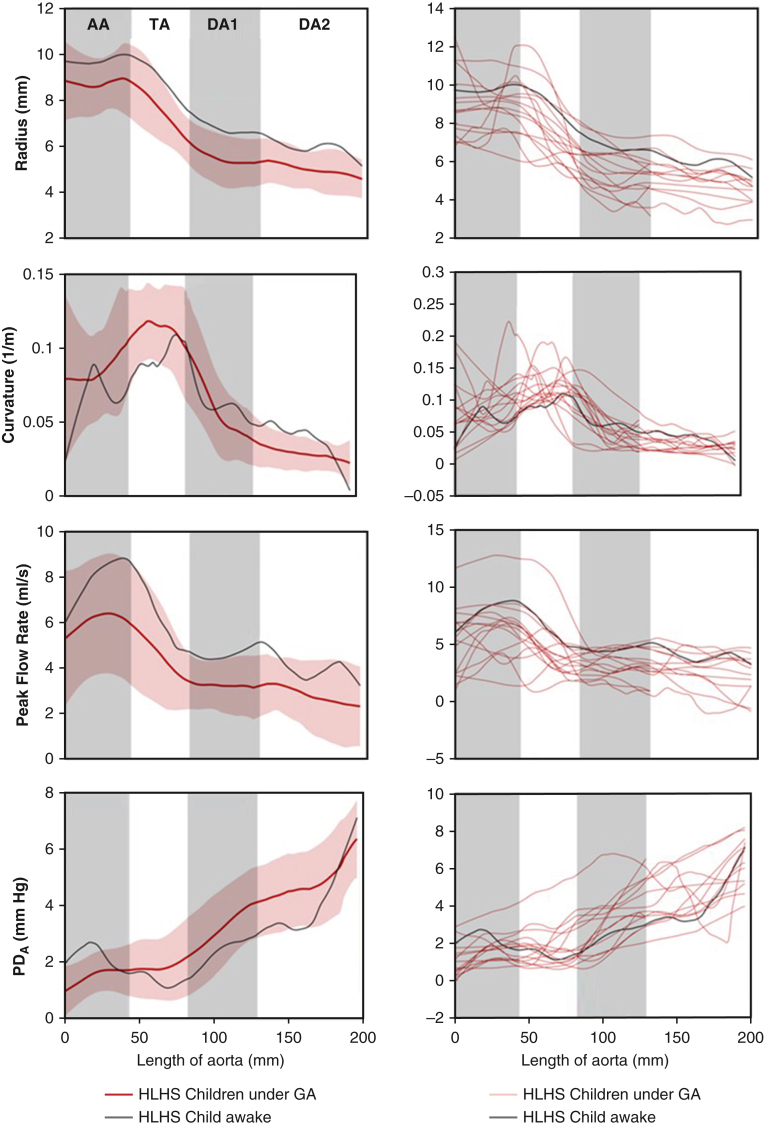
Comparative geometric and hemodynamic traces along aortic the center line for the cohort of hypoplastic left heart syndrome (HLHS) children under anesthesia during magnetic resonance imaging, including the respective mean ± standard deviation (*left side*) and individual traces (*right side*), and an additional awake HLHS child. The overall aortic length is the average length in the population. Each individual trace was fitted to the average length of each of 4 regions: ascending aorta (AA), transverse aortic arch (TA), and descending aorta proximal and distal aortas (DA1 and DA2).

**Figure E4:**
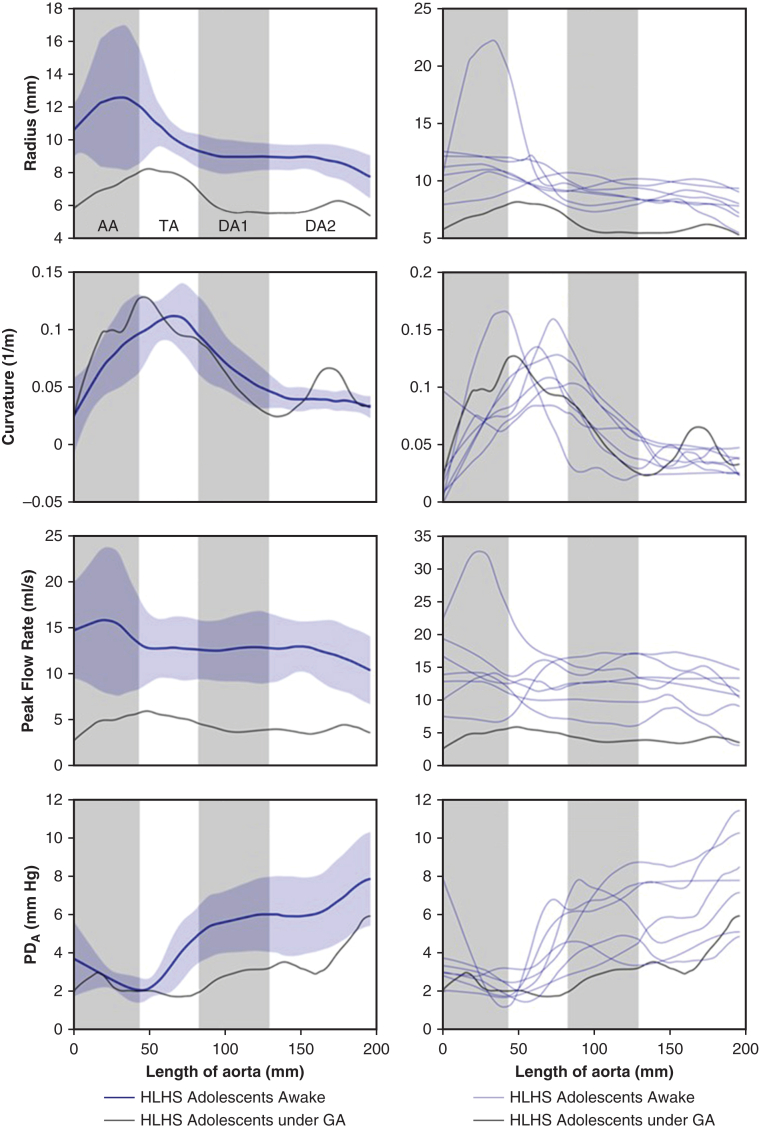
Comparative geometric and hemodynamic traces along the aortic center line for the awake hypoplastic left heart syndrome (HLHS) adolescent cohort during magnetic resonance imaging (MRI), including mean ± standard deviation (*left side*) and individual traces (*right side*), and an additional HLHS adolescent under anesthesia. The overall aortic length is the average length of the population. Each individual trace was fitted to the average length of each of 4 regions: ascending aorta (AA), transverse aortic arch (TA), and descending proximal and distal aortas (DA1 and DA2).

## Appendix E1

### Section 1: “Skate Park” Analogy of Inefficiencies

To illustrate the concepts of hemodynamic inefficiency more intuitively, we use the analogy of a “U-pipe” in a skate park. The heart is the elevator that brings the blood (ie, the skater) to the top of the U-pipe and increases its potential energy. When the valve opens, the skater accelerates down the U-pipe, transforming potential energy into blood flow momentum (ie, accelerating blood flow in space). Thus, maximum velocity occurs at the bottom of the U-pipe. When the skater starts to climb up on the other side of the U-pipe, the momentum is transformed back into potential energy, but not all of it is recovered; a part of it is dissipated by the friction of the wheels on the wall (ie, VD of blood).

In this analogy, the maximum momentum at the bottom of the U-pipe informs about the potential energy (ie, the pressure drop, the PD_A_) used to reach that maximum velocity. This PD_A_ is then a metric of the total energy loss that will occur by friction in the situation where the skater keeps rolling down the street until a full stop. Thus, it differs from metrics of WSS or VD that aim to directly measure the energy losses due to friction (which are challenging to quantify robustly in real-world data). This makes PD_A_ a robust surrogate measure of overall flow “inefficiency” (the greater the PD_A_, the greater the inefficiency), and why the pressure drop calculated by the Simplified Bernoulli equation from echocardiography is the metric used in clinical practice despite its simplifications and assumptions, as described previously.^14^

### Section 2: Brief Study of Metrics of Flow Inefficiency

**Rationale.** To illustrate the need for a more robust metric of flow inefficiency, we performed a brief analysis of how metrics of dissipation are affected by voxel resolution in 4D Flow datasets. We report on the 2 most prevalent metrics, cross-sectional VD and cross-sectional WSS and compare them to the proposed metric (PD_A_).^12^ A more thorough analysis and report of the limitations of these metrics can be found in the literature.^11^

**Methods.** We used an in silico model of a fully developed paraboloidal profile (the optimal flow configuration to reduce VD) in an idealized straight artery with a 10-mm radius and constant flow of 15 mL/s. These parameters are representative of the aortas of our infant population. This model uses the ground truth values for the metrics of inefficiency and enables analysis of the impact of image resolution, first assuming a high resolution of 9 voxels per radius and then down-sampling to 3 voxels per radius, representative of the data in our infant population.

**Results.** The in silico idealized flow profile illustrates that the PD_A_ quantification is robust to limited voxel resolutions, while WSS and VD are heavily damped and dependant on resolution (Figure 1, *A*). We also compared PD_A_, WSS, and VD values extracted from 4D Flow in selected patients to highlight the different roles of each biomarker (Figure 2, *A*). WSS was markedly lower in the least skewed profile (HLHS-5), in which high-velocity blood particles are concentrated in the middle of the vessel, away from the wall. Simplified Bernoulli is shown to underestimate pressure difference by relying on the peak velocity rather than on the full cross-sectional flow profile like in PD_A_.

### Section 3: Exploratory Result of the Impact of Anesthesia

**Problem statement.** Controlling for the impact of anesthesia in our results is challenging for many reasons. First, the use of anesthesia in children with HLHS is highly variable and complex in those with single ventricle, given that the patient must be asleep but simultaneously avoid bradycardia.^33^ And second, there are many interrelated physiologic aspects that lead to hemodynamic changes. As a reference, a study in aortic coarctation in young adults revealed that the change from rest to stress conditions increased the functional obstruction by ∼5-fold.^34^

**Exploratory study design.** Our retrospective study population did provide an opportunity to explore this problem. One subject from the HLHS adolescent was scanned with anesthesia (12.3 years old, heart rate 74 bpm) and 1 subject from the HLHS children group was scanned without anesthesia (10.1 years old, heart rate 139 bpm). These 2 subjects were eliminated from the main results, but allowed us to identify the relative changes in flow rate and PD_A_ compared to their other anesthesia group of the same age.

**Results.** The awake HLHS child exhibited similar heart rate and hemodynamic parameters (ie, flow rate and PD_A_) as the HLHS child under anesthesia (Figure 3, *A*). In contrast, the HLHS adolescent under anesthesia had a larger aortic diameter (ie, vasodilation) and a drop in hemodynamic markers owing to the relaxed state induced by anesthesia (Figure 4, *A*). Specifically, this case had a ∼3-fold smaller peak flow rate and ∼4-fold smaller ΔPD_A_ through the TA–DA1 transition. These 2 cases cover a wide spectrum of the impact of anesthesia, from no change to a factor of 4 in the PD_A_ metrics.

**Discussion.** These pilot results reflect the large variability in the impact of anesthesia in our results and suggest the capability of the vascular system to adapt to changes in flow demand.
